# Predicting neurological Adverse Drug Reactions based on biological, chemical and phenotypic properties of drugs using machine learning models

**DOI:** 10.1038/s41598-017-00908-z

**Published:** 2017-04-13

**Authors:** Salma Jamal, Sukriti Goyal, Asheesh Shanker, Abhinav Grover

**Affiliations:** 1 Department of Bioscience and Biotechnology, Banasthali University, Tonk, Rajasthan India; 2 Bioinformatics Programme, Centre for Biological Sciences, Central University of South Bihar, BIT Campus, Patna, Bihar India; 3 School of Biotechnology, Jawaharlal Nehru University, New Delhi, India

## Abstract

Adverse drug reactions (ADRs) have become one of the primary reasons for the failure of drugs and a leading cause of deaths. Owing to the severe effects of ADRs, there is an urgent need for the generation of effective models which can accurately predict ADRs during early stages of drug development based on integration of various features of drugs. In the current study, we have focused on neurological ADRs and have used various properties of drugs that include biological properties (targets, transporters and enzymes), chemical properties (substructure fingerprints), phenotypic properties (side effects (SE) and therapeutic indications) and a combinations of the two and three levels of features. We employed relief-based feature selection technique to identify relevant properties and used machine learning approach to generated learned model systems which would predict neurological ADRs prior to preclinical testing. Additionally, in order to explain the efficiency and applicability of the models, we tested them to predict the ADRs for already existing anti-Alzheimer drugs and uncharacterized drugs, respectively in side effect resource (SIDER) database. The generated models were highly accurate and our results showed that the models based on chemical (accuracy 93.20%), phenotypic (accuracy 92.41%) and combination of three properties (accuracy 94.18%) were highly accurate while the models based on biological properties (accuracy 82.11%) were highly informative.

## Introduction

Adverse drug reactions (ADRs) are unwanted phenotypic responses caused due to alterations in biological pathways in response to drug treatments^1^. Studies on ADRs have become more significant owing to the increasing number of morbidity and mortality due to severe ADRs. ADRs have been predicted as the fourth leading cause of death in the United States with a probability of 100 000 fatalities per year^2^. Using the fundamental drug discovery process, few amongst the thousands of lead compounds reach the clinical trials and actually make it to the market which involves billions of dollars and huge amount of time and labour. However even then most of the drugs fail in the phase IV clinical trials and in post marketing surveillance and the drug has a chance to be withdrawn due to ADRs^3^. These facts advocate the inevitable need for prediction of ADRs in early stages of drug discovery and development process.

In latest years, prediction of potential ADRs has become a research focus of utmost importance for a large number of pharmaceutical companies and a large number of studies have been conducted in this regard. The traditional method of ADRs prediction employed by these companies involved testing of the compounds by conducting biological assays which is an extremely challenging process in terms of time, effort, money and efficiency^4^. Recently a large number of studies have been reported which involve preclinical prediction of ADRs associated with drugs by integrating the side effects information^5^, protein targets, transporters and enzymes information^6^, chemical structure information^7^ and drugs therapeutic indications^2^.

Kanji *et al*.^8^ proposed a new strategy and generated a canonical correlation model for predicting side effects of drugs by combining their chemical properties with their target profiles. Zhang *et al*.^9^ used ensemble methods and devised feature selection based multi-label k-nearest neighbour method (FS-MLKNN) using which essential features for ADR prediction can be predicted. Huang *et al*. integrated drug information (drug target data and clinical observation data) with network information (protein-protein interaction networks and gene ontology information) and built *in silico* models for computer-aided ADR prediction of drugs^10^.

Although various methods have been proposed for prior prediction of ADRs for drugs, there still remains room for improvement. In the present era, there is an enormous amount of publicly available side effects data. This can serve significant if we could integrate it with chemical structure information, protein binding and therapeutic indication data. In this study, we have proposed a computational method in which we have integrated three levels of information, biological features (targets, transporters and enzymes), chemical information (PubChem substructure fingerprints) and phenotypic information (side effects and therapeutic indications) towards prediction of neurological ADRs. We have measured chemical similarity among the drugs and employed relief-based feature selection technique to identify features relevant for ADR prediction. To handle imbalance in the data, we have used Synthetic Minority Oversampling Technique (SMOTE)^11^ on train sets. These balanced training sets were used to generate *in silico* models which could predict neurological ADRs associated with drugs. Using SMOTE balanced training datasets, the machine learning models for each of the biological, chemical and phenotypic features as well as for combination of all the features for 22 neurological ADRs were generated. Furthermore, the models were employed to predict neurological side effects for uncharacterized drugs in SIDER for which no ADR information was available.

## Results and Discussion

**Figure 1:**
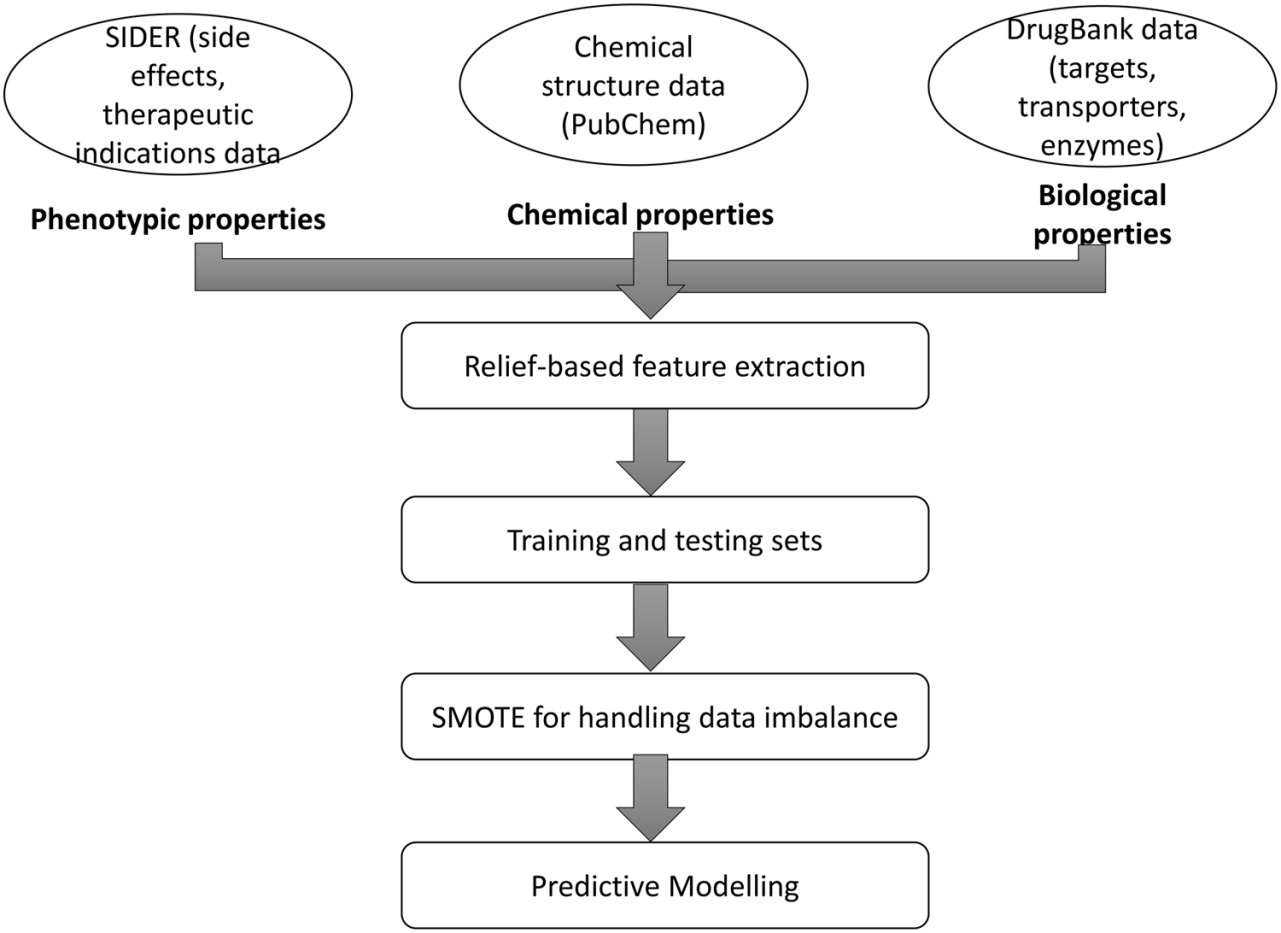
The computational methodology followed in the present study has been shown in Fig. 1.

The computational methodology followed in the present study has been shown in Fig. 1.

### Feature analysis

In order to remove the less important features with low significant contribution towards classification, reduce the dimensionality of the data and processing time, we used relief-based feature selection technique. The list of the features obtained after application of relief-based feature selection has been provided as Supplementary Table 1. Table 1 lists the number of features obtained after applying RemoveUseless filter and relief-based feature selection and types of features used to generate the models.

**Table 1:** Lists the number of features obtained after applying RemoveUseless filter and relief-based feature selection and types of features used to generate the models.

Type of feature		Initial number of features	RemoveUseless filter’	Relief-based selection	Total final features
Biological	Targets	954	945	52	78
	Transporters	87	86	13	
	Enzymes	168	165	13	
Chemical	Substructures	881	619	319	319
Phenotypic	Other ADRs	5462	5411	272	281
	Therapeutic indications	3046	1962	9	

### Models assessment

The performance of the models was evaluated using the testing dataset. Table 2 provides the list of the 22 neurological ADRs along with their SIDER ids for which the SMO models were generated.

**Table 2:** Provides the list of the 22 neurological ADRs along with their SIDER ids for which the SMO models were generated.

Neurological ADR	SIDER id
Arteritic anterior ischaemic optic neuropathy	C2242711
Autonomic neuropathy	C0259749
Nervous system disorder	C0007682
Neuralgia	C0040997
Neuritis	C0027813
Neuritis retrobulbar	C0085582
Neuroleptic malignant syndrome	C0027849
Neurologic reaction	C0235030
Neurological impairment	C0521654
Neurological symptom	C0235031
Neuromuscular block prolonged	C0520758
Neuromyopathy	C0027868
Neuropathy	C0442874
Neuropathy peripheral	C0031117
Neurosis	C0027932
Neurotoxicity	C0235032
Optic neuritis	C0029134
Peripheral motor neuropathy	C0235025
Peripheral sensorimotor neuropathy	C1112256
Peripheral sensory neuropathy	C0151313
Polyneuropathy	C0152025
Post herpetic neuralgia	C0032768

### Modelling using biological features

A total of 22 models were generated on a training set using a combination of 52 targets, 13 transporters and 13 enzymes totalling as 78 biological properties for 913 approved drugs. The models had an accuracy of 82.11%, a very high precision value of 0.94, and value for recall as 0.85, and F-score equal to 0.89. The model for ADR autonomic neuropathy came out to be the best predictive model having the highest accuracy value (98.9%), highest precision (0.99) and F-score of 0.99. Table 3 provides the performances of the models generated using the biological features.

**Table 3:** Provides the performances of the models generated using the biological features.

ADR event	Accuracy (%)	Precision	Recall	F-score	AUC
Arteritic anterior ischaemic optic neuropathy	71.58	0.99	0.72	0.83	0.36
Autonomic neuropathy	98.9	0.99	0.99	0.99	0.49
Nervous system disorder	59.56	0.6	0.86	0.7	0.55
Neuralgia	72.67	0.92	0.76	0.83	0.54
Neuritis	93.98	0.93	1	0.96	0.607
Neuritis retrobulbar	96.17	0.99	0.96	0.98	0.48
Neuroleptic malignant syndrome	57.37	0.97	0.56	0.71	0.63
Neurologic reaction	88.52	0.98	0.89	0.93	0.44
Neurological impairment	99.45	0.99	1	0.99	0.50
Neurological symptom	59.01	0.98	0.59	0.73	0.54
Neuromuscular block prolonged	92.89	0.99	0.93	0.96	0.46
Neuromyopathy	85.79	0.87	0.97	0.92	0.52
Neuropathy	69.39	0.88	0.74	0.80	0.559
Neuropathy peripheral	88.52	0.88	0.99	0.93	0.66
Neurosis	78.68	0.96	0.80	0.87	0.54
Neurotoxicity	79.23	0.95	0.82	0.88	0.41
Optic neuritis	80.87	0.94	0.84	0.89	0.42
Peripheral motor neuropathy	84.15	0.98	0.85	0.91	0.42
Peripheral sensorimotor neuropathy	97.81	0.99	0.98	0.98	0.49
Peripheral sensory neuropathy	73.77	0.97	0.74	0.84	0.57
Polyneuropathy	89.01	0.99	0.89	0.94	0.44
Post herpetic neuralgia	89.07	0.99	0.89	0.94	0.44

### Modelling using chemical features

The 22 machine learning models were generated using 319 PubChem chemical substructure fingerprints for 913 drugs. The models were highly informative having an accuracy of 93.20%, precision and recall value of 0.96 and 0.95 respectively, and F-score value equal to 0.95. As compared to the models trained using biological properties, these models were more predictive having greater mean value for all the parameters, indicating that the chemical structure played a significant role in drugs ADR prediction. Table 4 provides the performances of the models generated using the chemical features.

**Table 4:** Provides the performances of the models generated using the chemical features.

ADR event	Accuracy (%)	Precision	Recall	F-score	AUC
Arteritic anterior ischaemic optic neuropathy	98.90	0.99	0.99	0.99	0.49
Autonomic neuropathy	99.45	0.99	1	0.99	0.50
Nervous system disorder	64.48	0.67	0.73	0.70	0.63
Neuralgia	94.58	0.97	0.92	0.94	0.95
Neuritis	93.81	0.95	0.92	0.94	0.93
Neuritis retrobulbar	99.45	0.99	1	0.99	0.5
Neuroleptic malignant syndrome	91.25	0.95	0.94	0.95	0.62
Neurologic reaction	97.81	0.99	0.98	0.98	0.74
Neurological impairment	95.62	0.96	0.99	0.97	0.49
Neurological symptom	96.17	0.97	0.98	0.98	0.49
Neuromuscular block prolonged	100	1	1	1	1
Neuromyopathy	99.45	0.99	1	0.99	0.50
Neuropathy	69.64	0.86	0.77	0.81	0.49
Neuropathy peripheral	91.92	0.94	0.89	0.91	0.92
Neurosis	92.89	0.97	0.95	0.96	0.62
Neurotoxicity	92.34	0.97	0.94	0.96	0.68
Optic neuritis	89.07	0.95	0.92	0.94	0.52
Peripheral motor neuropathy	95.62	0.98	0.96	0.97	0.48
Peripheral sensorimotor neuropathy	99.45	0.99	1	0.99	0.50
Peripheral sensory neuropathy	95.02	0.97	0.97	0.97	0.48
Polyneuropathy	95.08	0.98	0.96	0.97	0.6
Post herpetic neuralgia	98.36	1	0.98	0.99	0.99

### Modelling using phenotypic features

Using 281 phenotypic properties which comprised 272 other SE and 9 indications, 22 SMO models were generated for 22 neurological ADRs. The models were very informative having accuracy of 92.41%, precision 0.97, recall value 0.93, and F-score 0.95. The models had similar performance when compared to modelled chemical features but had significantly high values (around 10% increase in accuracy) in comparison to the models with biological properties. Table 5 provides the performances of the models generated using the phenotypic features.

**Table 5:** Provides the performances of the models generated using the phenotypic features.

ADR event	Accuracy (%)	Precision	Recall	F-score	AUC
Arteritic anterior ischaemic optic neuropathy	99.45	0.99	1	0.99	0.50
Autonomic neuropathy	98.90	0.99	0.99	0.99	0.47
Nervous system disorder	87.43	0.92	0.84	0.88	0.87
Neuralgia	84.15	0.96	0.85	0.90	0.76
Neuritis	94.04	0.95	0.95	0.94	0.94
Neuritis retrobulbar	97.81	0.97	1	0.98	0.50
Neuroleptic malignant syndrome	90.71	0.96	0.93	0.95	0.66
Neurologic reaction	98.36	0.99	0.98	0.99	0.74
Neurological impairment	99.45	0.99	1	0.99	0.50
Neurological symptom	95.30	0.95	0.95	0.95	0.95
Neuromuscular block prolonged	98.90	0.99	0.99	0.99	0.49
Neuromyopathy	99.45	0.99	1	0.99	0.50
Neuropathy	74.31	0.92	0.76	0.83	0.67
Neuropathy peripheral	78.14	0.91	0.81	0.86	0.70
Neurosis	89.61	0.97	0.91	0.94	0.67
Neurotoxicity	85.24	0.98	0.86	0.91	0.71
Optic neuritis	83.06	0.93	0.88	0.9	0.54
Peripheral motor neuropathy	96.17	0.99	0.96	0.98	0.73
Peripheral sensorimotor neuropathy	99.45	0.99	1	0.99	0.50
Peripheral sensory neuropathy	90.71	0.98	0.91	0.95	0.75
Polyneuropathy	92.34	0.97	0.94	0.96	0.47
Post herpetic neuralgia	100	1	1	1	1

### Modelling using the combination of two levels of biological, chemical and phenotypic properties

We generated the models by the combining the two levels of features, chemical + phenotypic, biological + chemical, and phenotypic + biological. We observed that the combination of the two levels of features resulted in more accurate models, with chemical + phenotypic combination models being most accurate and extremely informative. The combined chemical + phenotypic properties models had an accuracy of 94.59%, precision value 0.96, recall 0.95, and F-score 0.96 (Table 6). The phenotypic + biological models also performed well having an accuracy of 92.96%, precision and recall value of 0.96 and 0.94 respectively, and F-score value 0.95 (Table 7). The combined biological + chemical models were least accurate among all three sets with accuracy 91.47%, precision, recall and F-score values equalling to 0.95%, 0.93% and 0.94%, respectively (Table 8).

**Table 6:** Provides the performances of the models generated using the Chemical + Phenotypic features.

ADR event	Accuracy (%)	Precision	Recall	F-score	AUC
Arteritic anterior ischaemic optic neuropathy	99.45	0.99	1	0.99	0.50
Autonomic neuropathy	99.45	0.99	1	0.99	0.50
Nervous system disorder	81.96	0.85	0.82	0.83	0.81
Neuralgia	86.88	0.93	0.91	0.92	0.62
Neuritis	88.52	0.94	0.93	0.93	0.61
Neuritis retrobulbar	99.45	0.99	1	0.99	0.50
Neuroleptic malignant syndrome	93.44	0.96	0.96	0.96	0.68
Neurologic reaction	98.9	0.99	0.99	0.99	0.74
Neurological impairment	99.45	0.99	1	0.99	0.50
Neurological symptom	96.72	0.97	0.98	0.98	0.49
Neuromuscular block prolonged	99.45	0.99	1	0.99	0.50
Neuromyopathy	99.45	0.99	1	0.99	0.50
Neuropathy	98.18	0.98	0.97	0.98	0.98
Neuropathy peripheral	76.5	0.87	0.83	0.85	0.61
Neurosis	92.89	0.97	0.94	0.96	0.68
Neurotoxicity	89.61	0.97	0.91	0.94	0.67
Optic neuritis	91.25	0.97	0.93	0.95	0.65
Peripheral motor neuropathy	98.36	0.98	0.99	0.99	0.49
Peripheral sensorimotor neuropathy	99.45	0.99	1	0.99	0.50
Peripheral sensory neuropathy	95.08	0.97	0.97	0.97	0.48
Polyneuropathy	97.26	0.97	0.99	0.98	0.49
Post herpetic neuralgia	99.45	1	0.99	0.99	0.99

**Table 7:** Provides the performances of the models generated using the Biological + Phenotypic features.

ADR event	Accuracy (%)	Precision	Recall	F-score	AUC
Arteritic anterior ischaemic optic neuropathy	99.45	0.99	1	0.99	0.50
Autonomic neuropathy	98.9	0.99	0.99	0.99	0.49
Nervous system disorder	84.69	0.89	0.82	0.86	0.85
Neuralgia	86.33	0.96	0.88	0.92	0.74
Neuritis	86.33	0.93	0.91	0.92	0.59
Neuritis retrobulbar	99.45	0.99	1	0.99	0.50
Neuroleptic malignant syndrome	94.53	0.97	0.97	0.97	0.75
Neurologic reaction	99.45	0.99	1	0.99	0.5
Neurological impairment	99.45	0.99	1	0.99	0.50
Neurological symptom	92.34	0.97	0.94	0.96	0.47
Neuromuscular block prolonged	98.9	0.99	1	0.99	0.49
Neuromyopathy	99.45	0.99	1	0.99	0.50
Neuropathy	75.4	0.91	0.78	0.84	0.66
Neuropathy peripheral	80.32	0.91	0.84	0.87	0.72
Neurosis	92.89	0.97	0.95	0.96	0.62
Neurotoxicity	88.52	0.97	0.9	0.93	0.66
Optic neuritis	90.16	0.96	0.93	0.94	0.59
Peripheral motor neuropathy	96.17	0.99	0.96	0.98	0.73
Peripheral sensorimotor neuropathy	91.8	0.98	0.92	0.95	0.76
Peripheral sensory neuropathy	99.45	0.99	1	0.99	0.5
Polyneuropathy	91.8	0.98	0.93	0.95	0.59
Post herpetic neuralgia	99.45	1	0.99	0.99	0.5

**Table 8:** Provides the performances of the models generated using the Biological + Chemical features.

ADR event	Accuracy (%)	Precision	Recall	F-score	AUC
Arteritic anterior ischaemic optic neuropathy	98.9	0.99	0.99	0.99	0.49
Autonomic neuropathy	99.45	0.99	1	0.99	0.50
Nervous system disorder	62.84	0.64	0.76	0.69	0.60
Neuralgia	83.6	0.92	0.89	0.90	0.54
Neuritis	85.79	0.94	0.89	0.92	0.62
Neuritis retrobulbar	99.45	0.99	1	0.99	0.50
Neuroleptic malignant syndrome	91.25	0.95	0.94	0.95	0.62
Neurologic reaction	98.36	0.99	0.98	0.99	0.74
Neurological impairment	99.45	0.99	1	0.99	0.50
Neurological symptom	94.53	0.97	0.96	0.97	0.48
Neuromuscular block prolonged	100	1.00	1.00	1.00	1.00
Neuromyopathy	99.45	0.99	1	0.99	0.50
Neuropathy	71.58	0.86	0.79	0.82	0.5
Neuropathy peripheral	71.58	0.88	0.75	0.81	0.62
Neurosis	91.25	0.96	0.94	0.95	0.54
Neurotoxicity	91.8	0.97	0.94	0.95	0.61
Optic neuritis	87.97	0.96	0.9	0.93	0.57
Peripheral motor neuropathy	97.26	0.98	0.98	0.98	0.49
Peripheral sensorimotor neuropathy	99.45	0.99	1	0.99	0.50
Peripheral sensory neuropathy	95.62	0.97	0.98	0.97	0.49
Polyneuropathy	94.53	0.97	0.96	0.97	0.48
Post herpetic neuralgia	98.36	1	0.98	0.99	0.99

### Modelling using the combined biological, chemical and phenotypic properties

The three levels of the features, biological (78), chemical (319) and phenotypic (291) were combined and a dataset of total 678 properties was created. The learned model systems generated had an accuracy value 94.18%, precision and recall corresponding to 0.96 and 0.96 respectively, and F-score value also 0.96. Table 9 provides the performances of the models generated using the combination of the three levels of features, biological, chemical, and phenotypic.

**Table 9:** Provides the performances of the models generated using the combination of the three levels of features, biological, chemical, and phenotypic.

ADR event	Accuracy (%)	Precision	Recall	F-score	AUC
Arteritic anterior ischaemic optic neuropathy	100	1	1	1	1
Autonomic neuropathy	99.45	0.99	1	0.99	0.50
Nervous system disorder	79.78	0.83	0.80	0.82	0.79
Neuralgia	85.24	0.93	0.89	0.91	0.61
Neuritis	90.71	0.94	0.95	0.95	0.62
Neuritis retrobulbar	99.45	0.99	1	0.99	0.50
Neuroleptic malignant syndrome	93.98	0.96	0.97	0.96	0.68
Neurologic reaction	99.45	0.99	1	0.99	0.75
Neurological impairment	99.45	0.99	1	0.99	0.50
Neurological symptom	96.17	0.97	0.98	0.98	0.49
Neuromuscular block prolonged	99.45	0.99	1	0.99	0.50
Neuromyopathy	99.45	0.99	1	0.99	0.50
Neuropathy	79.23	0.89	0.86	0.87	0.58
Neuropathy peripheral	81.42	0.89	0.88	0.88	0.67
Neurosis	93.98	0.97	0.96	0.96	0.69
Neurotoxicity	92.89	0.97	0.94	0.96	0.68
Optic neuritis	93.44	0.97	0.96	0.96	0.66
Peripheral motor neuropathy	97.81	0.98	0.98	0.98	0.49
Peripheral sensorimotor neuropathy	99.45	0.99	1	0.99	0.50
Peripheral sensory neuropathy	95.02	0.97	0.97	0.97	0.48
Polyneuropathy	96.72	0.98	0.98	0.98	0.61
Post herpetic neuralgia	99.45	1	0.99	0.99	0.99

### Case study on anti-Alzheimer drugs

In the present study, the three FDA approved drugs against Alzheimers, namely include Donepezil (DrugBank ID: DB00843), Galantamine (DrugBank ID: DB00674) and Memantine (DrugBank ID: DB01043), were removed before the generation of the models. The data for these three drugs was used as a control in order to assess the predictive capacity and performance of the models in addition to statistical analysis. As per the information derived from the SIDER database, Donepezil has been associated with the ADRs, Neuralgia and Nervous system disorder (NSD). The models for ADR Neuralgia and ADR NSD generated using chemical features predicted both the ADRs to be associated with Donepezil. SIDER lists Neuropathy peripheral (NP) and NSD as the side effects of Galantamine and the same was predicted by the NP and NSD models generated using the chemical, phenotypic and the combination of the three features. Memantine has been linked to all the three ADRs - Neuralgia, NSD and NP according to the SIDER database. However, ADR NSD modelled using phenotypic and combined features predicted NSD to be related to Memantine. ADR neuritis and optic neuritis was predicted to be associated with Donepezil by the optic neuritis model generated using biological, chemical and combined features. Various studies have reported the correlation between neuritis, optic neuritis and Alzheimers disease^12, 13^. The above results are clear indication of accuracy and the predictive ability of the generated models for 22 neurological ADRs.

### Prediction on drugs having no information in SIDER

To enhance the applicability of the generated SMO models for neurological ADRs, we predicted the ADRs for 103 DrugBank drugs having no information in SIDER. We found that all the models predicted NSD as one of the ADR associated with most of the drugs. The top ADRs associated with the drugs included NSD, neuralgia, neurotoxicity, neuroleptic malignant syndrome, peripheral sensory neuropathy and neuropathy. The biological properties NSD model predicted it to be linked to 45 drugs, the NSD model of chemical properties predicted it to be associated with 44 drugs and the combined feature NSD model found NSD to be connected with 15 drugs. No drugs were predicted to have neurological impairment (NI) as ADR except for 1 drug which was predicted by chemical features NI models.

To add relevance to our preliminary findings, we conducted an extensive literature search to find association between the drugs and side effects predicted by our models. According to a report by WHO library, Mefloquine was found to be related to various central nervous system adverse events which include major psychiatric disorders and symptoms, neurosis, neuropathies and various other neurological disorders^14^. High doses of cyanocobalamin are known to have possible associations with adverse neurological disorders^15^. Administration of quinolones might result in central nervous system events such as neurotoxicity and neurological ADRs have been ranked as second common group of ADRs associated with drugs of this class^16^. Serious central nervous system adverse events were found to be related to the drug, Sulindac^17^. Tetracyclines have been associated with neurotoxicity and neuromuscular blockage in addition to other neurotoxic events^18^. Irinotecan in combination with oxaliplatin induced various neurologic complications^19^, treatment with amiodarone induced polyneuropathy and other neurological complications^20^, severe axonal neuropathy and sensorimotor neuropathy was observed following treatment with arsenic trioxide^21^ and a 14.3% of serious neurological side effects were observed on administration of bromocriptine^22^. Mild neurologic adverse events were detected on treatment with docetaxel^23^, severe neuropsychiatric manifestations were found to be associated with azithromycin^24^, nitrofurantoin was reported to cause sensorimotor polyneuropathy when used in children^25^, cases of neurosensory adverse effects were observed on treatment with phenylbutazone^26^ and use of cocaine^27^, paclitaxel^28^ and tacrolimus^29^ is associated with severe neurotoxicity. Adverse neurological side effects and nervous system disorders were observed in mice on treatment with lopinavir^30^. A major life threatening neurological adverse event was observed in case of administration of vilazodone^31^.

### External dataset validation

Considering the applicability domain as well as performance of the generated models, the machine learning models were evaluated on 16383 MyriaScreen compounds obtained from Sigma-Aldrich. The most common side effects predicted include neuropathy peripheral, NSD, neuralgia, neuritis, neuropathy and neuroleptic malignant syndrome. NSD was predicted for 1280 compounds by the combined properties model and 6843 compounds by the chemical properties model. NMS was predicted for all the compounds by biological features model, for 344 compounds by the combined features model and 953 compounds by the chemical features model. The ADR which were not predicted to be associated with any of the compounds include autonomic neuropathy, neuromuscular block prolonged and neurological impairment. The results were very similar to the results obtained on testing the models on the uncharacterized drugs having no side effect predicted in SIDER.

## Discussion

The present study proposes a rigorous, exhaustive and integrative computational protocol to generate machine learning models using biological, chemical and phenotypic properties of the drugs for the prediction of neurological ADRs. In this study, a total of 176 machine learning SMO models were generated using biological (targets, transporters and enzymes), chemical (substructures), phenotypic (SE and indications) properties for 22 neurological ADRs. To find the most important and quality attributes, we employed relief-based feature selection algorithm using which the complexity of the dataset reduced in addition to the computational time involved. We further employed SMOTE method on the training set to handle the imbalance in the dataset which performs by generating synthetic examples of the minority class. Among the three types of features and their combination, the phenotypic features data appeared to be most informative followed by chemical features as compared to the biological features. Upon addition of the chemical and phenotypic data to the biological data, the performance of the models significantly improved with accuracy from 82.11 to 94.18, recall from 0.85 to 0.96 and f-score from 0.89 to 0.96. However, the overall performances of the models generated using the three levels of features was similar to the chemical and phenotypic features alone. This denotes that chemical and phenotypic data of drugs were most predictive for ADR prediction. We also generated the models using the combination of two levels of features, chemical + phenotypic, biological + chemical, and phenotypic + biological. We observed that the combination models performed better than the models generated using one type of feature, with chemical + phenotypic properties models being the most accurate.

Furthermore, to prove the predictive power and to validate the accuracy of the generated models, the models were tested on anti-Alzheimer drugs and on the drugs with no SE information available in the SIDER database. We found that the generated models were highly accurate and predictive. Overall, the present study clearly delineates the potential of data integration approaches in predicting clinically important ADRs prior to the clinical trials.

## Methodology

### Data extraction and dataset construction

The present study was performed on the approved drugs obtained from DrugBank^32^ database which is a freely accessible comprehensive bioinformatics resource of drugs, their targets, structure and pathways.

### Side-effect datasets

The information about the drug side-effects was obtained from SIDER^4^ database version 4.1. SIDER (side effect resource) is a publicly available resource that contains information about the medicines existing in the market place and their recorded ADRs. As of October 2015, SIDER includes information about 1430 drugs and 5868 side effect keywords. In the present study, the entire SIDER database was downloaded and information about side effects was extracted. SIDER employs STITCH compound ids from which PubChem compound IDs (CID) can be obtained as mentioned in this rule (ftp://xi.embl.de/SIDER/2015-10-21/, Accessed April 2, 2016). The 1991 approved drugs obtained from DrugBank were mapped to the SIDER database using PubChem CIDs and the corresponding side-effects and therapeutic indications were obtained directly. A total of 933 drugs were successfully mapped to their respective DrugBank Ids which constituted the final dataset of 933 drugs, 5462 SE and 3046 therapeutic indications. Finally, each of the 933 drugs was represented as a binary matrix, the elements of which encoded the presence or absence of each of the 5462 SE and 3046 therapeutic indications. In each of 5462 and 3046 dimensional binary matrix, the entry 1 indicated the presence of the SE or therapeutic indication whereas the entry 0 indicated their absence.

### Chemical structure dataset

After mapping to the SIDER database, we obtained the chemical structure information for 933 drugs and used PaDEL^33^ software to generate the PubChem^34^ substructure fingerprints resulting in 881 chemical substructure fingerprints for 928 drugs. To this end, we had an 881 dimensional binary matrix, the elements, 1 or 0, of which corresponded to the presence or absence of the corresponding fingerprint respectively, for each of the 928 drugs.

### DrugBank data

The final 928 approved drugs were mapped to the DrugBank database from which information about the protein targets, transporters and enzymes was directly retrieved. To obtain such information, the DrugBank provided UniProt^35^ IDs were used and we extracted information about 954 protein targets, 87 transporters and 168 enzymes. As mentioned for the chemical structure dataset, we had a binary matrix the elements of which were either 1 or 0 indicating the presence or absence of a particular target (954), transporter (87) or enzyme (168) respectively, for each of the 928 approved drugs.

In conclusion, the phenotypic properties of the 928 drugs consisted of SE and therapeutic indications obtained from SIDER, the chemical properties were denoted by the PubChem fingerprints and the biological properties were constituted by drug protein targets, transporters and enzymes. Finally, in the resulting comma separated value (csv) files consisting of biological, chemical, phenotypic and the combination of the three features, a column named Outcome was appended which had a ‘Yes’ or ‘No’ value if a particular SE was associated with a drug or not.

### Chemical structure similarity measurement

We computed Tanimoto coefficient (TC) between the drugs using the ChemmineR package available from R scripting language^36^. ChemmineR converts the chemical structures in the Structural Data Format (SDF) to atom pair fingerprints and the obtained fingerprints are used for the similarity calculation. The drug chemical structures having Tanimoto similarity coefficient greater than 0.75 cut-off were considered as structurally similar drugs and were removed from the dataset resulting in the final set of 926 drugs.

### Relief-based features extraction

The drug molecules having uniform values for all the features, biological, chemical and phenotypic were removed using the RemoveUseless filter available in Weka^37^, which is a machine learning platform. The resultant dataset was then split into 80% training set and 20% test set using a custom Perl script, where training data was used for generation of predictive models and the test set was used for the model evaluation purpose. While performing feature selection the test set was used as a complete held-out data and feature selection was performed on the training sets to remove any biasness and post that the models were generated using train sets and were evaluated on the test sets.

Further, relief-based feature selection technique from Weka in combination with ranker search was employed to identify the features contributing significantly towards the ADR prediction task. The feature selection process also reduces the complexity of the dataset and the processing time required. ReliefAttributeEval is one of the most successful and widely used technique for evaluating the features based on their quality^38^. The algorithm assesses the effectiveness of a feature by repeated sampling of an instance and considers the value of the given feature based on the one-nearest-neighbour classifier^39^. The basic idea of relief feature selection algorithm is that it repetitively estimates the weights for features of an instance on the basis of their capability of discrimination amongst neighbouring instances. The weight for the feature decreases if it differs from the same feature in neighbouring instances of the same class more than neighbouring instances of the other class. After various iterations, the feature with the relevance greater than the threshold is selected^38^. Ranker search method was used along with ReliefAttributeEval which ranks the features based on their individual evaluations.

We investigated the other feature selection algorithms which include a gain-ratio based attribute evaluation, oneR algorithm, chi-square based selection, filtered attribute evaluator, information gain-based attribute evaluation and best first attribute selection, to select the important attributes. However, most of these feature selection algorithms gave same ranking to all the attributes as we obtained in case of relief-based selection. Few of the selection algorithms did not give any ranking to the features. The BestFirst method gave 9 biological features, 12 phenotypic and 4 chemical features as significantly relevant which was very less number of attributes resulting in discarding almost all of the features. Thus the feature selection, in the present study, was carried out at two levels, initially using RemoveUseless algorithm followed by relief-based feature selection.

### SMOTE for handling data imbalance

A dataset is considered as imbalanced if one class is over-represented while the other class is under-represented. Since not all the drugs were associated with many SE, this resulted in a highly imbalanced dataset and to introduce a balance between the majority and minority class, SMOTE^11^ method available from Weka was used on the training sets. SMOTE is an oversampling technique in which the under-sampled or the minority class is balanced by creation of synthetic examples and the data is resampled. The minority class is over-sampled by taking each instance of this class and computing Euclidean instance within the k-nearest members of the minority class and then introducing synthetic instances. The neighbouring instances from k-nearest neighbours are chosen randomly depending upon the amount of over-sampling required. In the present study the number of nearest neighbours’ value was kept as default which is 5. To generate the synthetic examples, the difference between the input vector under consideration and its nearest neighbour is multiplied by a random number and added to the input vector under consideration^40^. Table 10 provides the information about the number of instances obtained after applying SMOTE for each of the 22 neurological ADRs. Supplementary Table 2 mentions the different percentages at which the under-sampled class was over-sampled using SMOTE method.

**Table 10:** Provides the information about the number of training data instances obtained after applying SMOTE for each of the 22 neurological ADRs.

Neurological ADR	SMOTE instances		No. of instances before applying SMOTE	
	Training data (Positive outcome Yes)	Training data (Negative outcome No)	Training data (Positive outcome Yes)	Training data (Negative outcome No)
Arteritic anterior ischaemic optic neuropathy	628	730	3	730
Autonomic neuropathy	628	731	2	731
Nervous system disorder	612	731	2	731
Neuralgia	627	675	57	676
Neuritis	616	676	56	677
Neuritis retrobulbar	628	731	2	731
Neuroleptic malignant syndrome	640	693	40	693
Neurologic reaction	660	728	2	731
Neurological impairment	628	731	2	731
Neurological symptom	644	718	14	719
Neuromuscular block prolonged	612	731	731	2
Neuromyopathy	628	731	731	2
Neuropathy	576	637	96	637
Neuropathy peripheral	585	616	117	616
Neurosis	702	706	27	706
Neurotoxicity	702	706	27	706
Optic neuritis	609	705	29	704
Peripheral motor neuropathy	594	724	3	730
Peripheral sensorimotor neuropathy	628	731	2	731
Peripheral sensory neuropathy	620	713	20	713
Polyneuropathy	656	717	16	717
Post herpetic neuralgia	628	730	3	730

Additionally, we have generated the models using the imbalanced data as input without applying SMOTE technique. The results obtained have been provided as Supplementary Table 3. We would like to report that we obtained very similar results for all the generated models using all the types of features, biological, chemical, phenotypic and merged.

### Predictive modelling

During the generation of predictive models, the neurological ADRs prediction task was treated as a binary classification problem where each drug molecule was considered to either cause a particular ADR (labelled Yes) or not (labelled No). For biological, chemical, phenotypic and combined features for 22 neurological ADRs, a total of 176 predictive classifier models were generated using Sequential Minimization Algorithm (SMO), an implementation of Support Vector Machines (SVM), available from Weka. SVM have been widely used for the classic binary classification problems owing to their capability of handling large training sets as well as generally faster computation time^41–43^. The algorithm operates in an iterative manner by breaking the large quadratic problem (QP) into a range of smaller sub-QPs which are further solved in a systematic mode^44^. SVM is a discriminative classifier which uses an optimal hyperplane separating the new instances and further categorizing them. The SVM algorithm finds a hyperplane that separates the positive instances with negative ones and gives maximum distance between the two classes by creating a gap as wide as possible. This is the case of the linear classification problem, however, in addition, SVM uses kernel method that transforms non-linear space into linear ones for non-linear classification^44^. Default parameters were used for SMO which include Polykernel as the kernel type with complexity parameter, c-value equal to 1.0 to build the models. The predictive models were generated using the SMOTE balanced training set and 10-fold cross validation was used in the present study.

### Evaluation measures for predictive models

A total of 176 machine learning models were generated for 22 neurological ADRs which were evaluated using receiver operating characteristic (ROC), accuracy, precision, recall and F-measure. ROC curve is a graphical plot of true positive rate (or sensitivity or recall) vs false positive rate (1-specificity). True positive rate (TPR = TP/(TP + FN)) is the proportion of correctly identified positives while false positive rate is the proportion of correctly identified negatives. Accuracy (Q) is the proportion of correctly identified instances (Q = TP + TN/(TP + TN + FP + FN)). Precision (P) is the fraction of correctly identified positives against all the predicted positives (P = TP/(TP + FP)). The performance for the 176 models for 22 neurological ADRs was averaged for each of the class of the properties, biological, chemical, phenotypic and a combination of all the three properties.

## Electronic supplementary material

Supplementary information
