# Supplementary material for: Predicting neurological Adverse Drug Reactions based on biological, chemical and phenotypic properties of drugs using machine learning models
Source: Sci Rep. 2017 Apr 13;7:872. doi: 10.1038/s41598-017-00908-z (PMC5429831; doi:10.1038/s41598-017-00908-z)
Supplement: Supplementary file 1 — Supplementary information [file 41598_2017_908_MOESM1_ESM.pdf]

Predicting neurological Adverse Drug Reactions based on biological, chemical and phenotypic properties of drugs using machine learning models

Salma Jamal[a], Sukriti Goyal[a], Asheesh Shanker[a, b], Abhinav Grover[c*]

[a] Department of Bioscience and Biotechnology, Banasthali University, Tonk, Rajasthan, India

[b]Bioinformatics Programme, Centre for Biological Sciences, Central University of South Bihar, BIT Campus, Patna, Bihar, India

[c] School of Biotechnology, Jawaharlal Nehru University, New Delhi, India

*Corresponding author: abhinavgr@gmail.com, agrover@jnu.ac.in

Supplementary table 1 lists the features obtained after application of relief-based feature selection.

Biological		Phenotypic	Chemical
P24462	enzymes	C0919572	PubchemFP518
P20815		C1319200	PubchemFP148
P08684		C0281803	PubchemFP149
P10635		C0524662	PubchemFP336
P11712		C0919569	PubchemFP517
P05177		C0235524	PubchemFP422
P33261		C0857494	PubchemFP515
P10632		C0858765	PubchemFP300
P20813		C0860713	PubchemFP615
P05181		C0020440	PubchemFP3
P04798		C0231837	PubchemFP636
P11509		C1141861	PubchemFP682
P06276		C1619712	PubchemFP709
P23219	targets	C1510472	PubchemFP654
P35354		C0026650	PubchemFP688
P35348		C0036973	PubchemFP560
P08913		C0162297	PubchemFP430
P11229		C0026837	PubchemFP16
P04150		C0027066	PubchemFP593
P14416		C0206277	PubchemFP375
P14867		C0235063	PubchemFP657
P34903		C0855775	PubchemFP585
P47869		C0023066	PubchemFP646
P35367		C0011615	PubchemFP377
P31644		C0151828	PubchemFP600
Q16445		C0003578	PubchemFP356
P48169		C0013384	PubchemFP665
Q8N1C3		C0019521	PubchemFP495
P47870		C0497247	PubchemFP546
Q99928		C0235146	PubchemFP611
Q9UN88		C0233407	PubchemFP502
O14764		C0547030	PubchemFP569
P18507		C0340865	PubchemFP545
P78334		C0018790	PubchemFP439
P28472		C0006266	PubchemFP376
O00591		C0014591	PubchemFP645
P18505		C0013144	PubchemFP659
P28223		C0009782	PubchemFP437
P35368		C3665347	PubchemFP180
P07550		C0428977	PubchemFP628

P20309		C0015397	PubchemFP680
P08172		C0025061	PubchemFP633
P28335		C0018799	PubchemFP566
P08908		C0042373	PubchemFP449
P18089		C0004936	PubchemFP451
P08588		C0021053	PubchemFP689
P35372		C0085593	PubchemFP696
P18825		C0011991	PubchemFP549
P23975		C0003811	PubchemFP181
P21728		C0041657	PubchemFP341
P31645		C0041834	PubchemFP405
P41143		C0085631	PubchemFP299
P28221		C0010200	PubchemFP607
P28222		C0178298	PubchemFP393
P11388		C0344232	PubchemFP464
P08173		C0000737	PubchemFP579
P25100		C0687713	PubchemFP573
P03372		C0027765	PubchemFP143
P08912		C0020538	PubchemFP698
P41145		C0009676	PubchemFP619
P35462		C0037274	PubchemFP684
Q72547		C0036572	PubchemFP346
P10275		C0232462	PubchemFP578
Q14524		C0013395	PubchemFP603
Q01959		C0020458	PubchemFP618
P08183	transporters	C0009806	PubchemFP641
Q4U2R8		C0039231	PubchemFP552
Q9UNQ0		C0948594	PubchemFP668
P46059		C0917801	PubchemFP634
Q16348		C0002792	PubchemFP584
O15245		C0013404	PubchemFP677
P46721		C2830004	PubchemFP608
O15244		C0003123	PubchemFP595
Q92887		C0040034	PubchemFP520
Q9Y6L6		C0030554	PubchemFP470
Q8TCC7		C0231528	PubchemFP371
P33527		C0043352	PubchemFP391
O76082		C0020649	PubchemFP308
		C0017178	PubchemFP655
		C0036974	PubchemFP406
		C0231218	PubchemFP535
		C0003862	PubchemFP570
		C0015672	PubchemFP548
		C2047937	PubchemFP574

		C0003467	PubchemFP637
		C0030252	PubchemFP384
		C0009450	PubchemFP418
		C0039070	PubchemFP366
		C0008031	PubchemFP370
		C0234215	PubchemFP692
		C1443060	PubchemFP257
		C0233494	PubchemFP664
		C0023530	PubchemFP442
		C0027769	PubchemFP564
		C0004604	PubchemFP453
		C0013604	PubchemFP256
		C0040822	PubchemFP355
		C0002871	PubchemFP697
		C0042571	PubchemFP358
		C0016204	PubchemFP387
		C0037763	PubchemFP704
		C0030193	PubchemFP255
		C0040264	PubchemFP15
		C0002170	PubchemFP396
		C0002994	PubchemFP538
		C0035455	PubchemFP541
		C0162429	PubchemFP381
		C0031350	PubchemFP606
		C0015967	PubchemFP498
		C0041969	PubchemFP651
		C0042075	PubchemFP403
		C0085649	PubchemFP506
		C0014742	PubchemFP472
		C0042109	PubchemFP482
		C0037199	PubchemFP656
		C0013378	PubchemFP617
		C0242350	PubchemFP643
		C0042023	PubchemFP443
		C0162830	PubchemFP340
		C0018524	PubchemFP683
		C0038362	PubchemFP523
		C0001824	PubchemFP382
		C0851353	PubchemFP186
		C0009763	PubchemFP678
		C0019158	PubchemFP660
		C0267792	PubchemFP640
		C0032285	PubchemFP555
		C0038325	PubchemFP613

		C0019080	PubchemFP380
		C0043094	PubchemFP666
		C0042029	PubchemFP594
		C0518015	PubchemFP179
		C1384353	PubchemFP12
		C0851341	PubchemFP592
		C0004093	PubchemFP542
		C0020580	PubchemFP553
		C0018965	PubchemFP710
		C0020456	PubchemFP524
		C0027947	PubchemFP712
		C0043096	PubchemFP392
		C0006277	PubchemFP604
		C0013428	PubchemFP599
		C0948089	PubchemFP185
		C0027051	PubchemFP540
		C0855242	PubchemFP556
		C0017152	PubchemFP490
		C0038990	PubchemFP530
		C0011124	PubchemFP516
		C0011168	PubchemFP539
		C0085633	PubchemFP434
		C0022346	PubchemFP20
		C0233472	PubchemFP614
		C0149931	PubchemFP146
		C0035078	PubchemFP420
		C0003864	PubchemFP431
		C0004134	PubchemFP671
		C0030305	PubchemFP708
		C0016382	PubchemFP390
		C0002962	PubchemFP452
		C0151786	PubchemFP626
		C0021400	PubchemFP589
		C0011175	PubchemFP565
		C0030312	PubchemFP345
		C0029408	PubchemFP476
		C0004096	PubchemFP357
		C0031117	PubchemFP365
		C0080274	PubchemFP440
		C0020651	PubchemFP576
		C0039971	PubchemFP351
		C0232461	PubchemFP450
		C0002622	PubchemFP521
		C0497156	PubchemFP597

		C0151905	PubchemFP446
		C0034150	PubchemFP285
		C0151827	PubchemFP679
		C0014518	PubchemFP192
		C0011570	PubchemFP487
		C0013456	PubchemFP339
		C0542571	PubchemFP379
		C0014457	PubchemFP145
		C0404521	PubchemFP699
		C0006145	PubchemFP373
		C1306341	PubchemFP441
		C0041912	PubchemFP491
		C0026826	PubchemFP333
		C0017160	PubchemFP14
		C1368065	PubchemFP484
		C0013491	PubchemFP674
		C3665386	PubchemFP447
		C0012569	PubchemFP416
		C0011053	PubchemFP493
		C0033975	PubchemFP519
		C0042267	PubchemFP596
		C0232849	PubchemFP528
		C0013595	PubchemFP571
		C0014724	PubchemFP2
		C0032617	PubchemFP818
		C0151908	PubchemFP567
		C0149745	PubchemFP335
		C0151904	PubchemFP389
		C0022408	PubchemFP623
		C0010692	PubchemFP577
		C0020625	PubchemFP798
		C0042024	PubchemFP504
		C0403632	PubchemFP572
		C0349506	PubchemFP797
		C0233632	PubchemFP445
		C0000731	PubchemFP777
		C0424295	PubchemFP582
		C0014868	PubchemFP662
		C0243026	PubchemFP338
		C0520966	PubchemFP352
		C0235431	PubchemFP776
		C0017181	PubchemFP667
		C1167965	PubchemFP293
		C0025222	PubchemFP353

		C3263723	PubchemFP620
		C0851354	PubchemFP632
		C0007859	PubchemFP33
		C0234458	PubchemFP372
		C0700590	PubchemFP672
		C0575081	PubchemFP691
		C0015371	PubchemFP435
		C0027441	PubchemFP178
		C0009319	PubchemFP819
		C0011253	PubchemFP711
		C0023218	PubchemFP638
		C0231530	PubchemFP334
		C0018099	PubchemFP386
		C0233471	PubchemFP860
		C0026821	PubchemFP821
		C1565489	PubchemFP438
		C0020615	PubchemFP499
		C0085632	PubchemFP690
		C0024902	PubchemFP547
		C0011849	PubchemFP432
		C0006840	PubchemFP485
		C0151766	PubchemFP286
		C0020443	PubchemFP580
		C0038454	PubchemFP144
		C0002878	PubchemFP19
		C0018418	PubchemFP758
		C0152459	PubchemFP536
		C0002453	PubchemFP735
		C0852911	PubchemFP501
		C0020621	PubchemFP362
		C1257843	PubchemFP550
		C0746883	PubchemFP702
		C0338831	PubchemFP598
		C0028084	PubchemFP639
		C0162835	PubchemFP591
		C0235660	PubchemFP378
		C0151514	PubchemFP503
		C0011551	PubchemFP342
		C0392156	PubchemFP507
		C0027651	PubchemFP383
		C0234133	PubchemFP663
		C0030196	PubchemFP586
		C0018801	PubchemFP531
		C0023067	PubchemFP412

		C0017601	PubchemFP714
		C0017979	PubchemFP294
		C0267596	PubchemFP681
		C0549249	PubchemFP37
		C0086439	PubchemFP839
		C0423791	PubchemFP602
		C0085932	PubchemFP658
		C0002874	PubchemFP840
		C0263449	PubchemFP701
		C0026961	PubchemFP861
		C2225524	PubchemFP693
		C0020039	PubchemFP673
			PubchemFP182
			PubchemFP703
			PubchemFP11
			PubchemFP385
			PubchemFP532
			PubchemFP259
			PubchemFP705
			PubchemFP624
			PubchemFP581
			PubchemFP642
			PubchemFP184
			PubchemFP590
			PubchemFP183
			PubchemFP700
			PubchemFP489
			PubchemFP500
			PubchemFP756
			PubchemFP601
			PubchemFP397
			PubchemFP349
			PubchemFP824
			PubchemFP755
			PubchemFP661
			PubchemFP563
			PubchemFP131
			PubchemFP621
			PubchemFP337
			PubchemFP652
			PubchemFP480
			PubchemFP533
			PubchemFP368
			PubchemFP132

			PubchemFP18
			PubchemFP761
			PubchemFP544
			PubchemFP610
			PubchemFP462
			PubchemFP537
			PubchemFP465
			PubchemFP779
			PubchemFP258
			PubchemFP630
			PubchemFP129
			PubchemFP473
			PubchemFP475
			PubchemFP21
			PubchemFP483

Supplementary table 2 mentions the different percentages at which the under-sampled class was over-sampled using SMOTE method.

**Neurological ADR**	**SMOTE % (final %age used is in bold)**					
Arteritic anterior ischaemic optic neuropathy	100	150	200	250	**300**	
Autonomic neuropathy	50	100	150	200	250	**300**
Neuromuscular block prolonged	50	100	150	200	250	**290**
Neuralgia	**1000**					
Neuritis	**1000**					
Neuropathy	**500**					
Neuromyopathy	50	100	150	200	250	**300**
Neurologic reaction	1000	**1100**				
Neurosis	**2500**					
Neurotoxicity	**2500**					
Neurological impairment	50	100	150	200	250	**300**
Neuroleptic malignant syndrome	**1500**					
Neuropathy peripheral	**400**					
Neuritis retrobulbar	**300**					
Neurological symptom	**4500**					
Nervous system disorder	**Balanced**					
Optic neuritis	**2000**					
Post herpetic neuralgia	100	150	200	250	**300**	
Peripheral motor neuropathy	**6500**					
Polyneuropathy	**4000**					
Peripheral sensory neuropathy	**3000**					
Peripheral sensorimotor neuropathy	50	100	150	200	250	**300**

Supplementary table 3 provides the performances of the models generated using the three levels of features, biological, chemical, and phenotypic and their combination using the imbalanced data as input without applying SMOTE technique.

Modelling using Biological features

	Accuracy (%)	Precision	Recall	F-score	AUC
Arteritic anterior ischaemic optic neuropathy	99.45	0.99	1	0.99	0.50
Autonomic neuropathy	99.45	0.99	1	0.99	0.50
Nervous system disorder	59.5	0.60	0.86	0.70	0.55
Neuralgia	91.25	0.91	0.99	0.95	0.49
Neuritis	93.98	0.93	1	0.96	0.6
Neuritis retrobulbar	91.8	0.92	0.99	0.95	0.49
Neuroleptic malignant syndrome	94.5	0.94	1	0.97	0.50
Neurologic reaction	98.9	0.98	1	0.99	0.50
Neurological impairment	99.45	0.99	1	0.99	0.50
Neurological symptom	97.8	0.97	1	0.98	0.50
Neuromuscular block prolonged	99.45	0.99	1	0.99	0.50
Neuromyopathy	99.45	0.99	1	0.99	0.50
Neuropathy	86.88	0.86	1	0.93	0.50
Neuropathy peripheral	83.60	0.83	1	0.91	0.50
Neurosis	96.17	0.96	1	0.98	0.50
Neurotoxicity	96.17	0.96	1	0.98	0.50
Optic neuritis	95.62	0.95	1	0.97	0.50
Peripheral motor neuropathy	98.90	0.98	1	0.99	0.50
Peripheral sensorimotor neuropathy	99.45	0.99	1	0.99	0.50
Peripheral sensory neuropathy	97.26	0.97	1	0.98	0.50
Polyneuropathy	97.81	0.97	1	0.98	0.50
Post herpetic neuralgia	99.45	0.99	1	0.99	0.50

Modelling using Chemical features

	Accuracy (%)	Precision	Recall	F-score	AUC
Arteritic anterior ischaemic optic neuropathy	98.90	0.99	0.99	0.99	0.49
Autonomic neuropathy	99.45	0.99	1	0.99	0.50
Nervous system disorder	64.48	0.67	0.73	0.70	0.63
Neuralgia	89.61	0.92	0.97	0.94	0.51
Neuritis	90.16	0.92	0.97	0.94	0.52
Neuritis retrobulbar	99.45	0.99	1	0.99	0.5
Neuroleptic malignant syndrome	94.53	0.96	0.97	0.97	0.68
Neurologic reaction	98.36	0.98	0.99	0.99	0.49
Neurological impairment	98.90	0.99	0.99	0.99	0.49
Neurological symptom	96.17	0.97	0.98	0.98	0.49
Neuromuscular block prolonged	99.45	0.99	1	0.99	0.50
Neuromyopathy	99.45	0.99	1	0.99	0.50
Neuropathy	78.14	0.87	0.88	0.87	0.50
Neuropathy peripheral	75.40	0.85	0.85	0.85	0.54
Neurosis	94.53	0.96	0.97	0.97	0.56
Neurotoxicity	92.89	0.97	0.95	0.96	0.62
Optic neuritis	93.44	0.96	0.97	0.96	0.54
Peripheral motor neuropathy	98.36	0.98	0.99	0.99	0.49
Peripheral sensorimotor neuropathy	99.45	0.99	1	0.99	0.50
Peripheral sensory neuropathy	96.17	0.97	0.98	0.98	0.49
Polyneuropathy	96.17	0.97	0.98	0.98	0.49
Post herpetic neuralgia	98.90	1	0.98	0.99	0.99

Modelling using Phenotypic features

	Accuracy (%)	Precision	Recall	F-score	AUC
Arteritic anterior ischaemic optic neuropathy	99.45	0.99	1	0.99	0.50
Autonomic neuropathy	98.36	0.99	0.98	0.99	0.49
Nervous system disorder	87.43	0.92	0.84	0.88	0.87
Neuralgia	88.52	0.93	0.94	0.93	0.57
Neuritis	89.07	0.92	0.96	0.94	0.48
Neuritis retrobulbar	99.45	0.99	1	0.99	0.50
Neuroleptic malignant syndrome	92.89	0.94	0.97	0.96	0.53
Neurologic reaction	99.45	0.99	1	0.99	0.75
Neurological impairment	99.45	0.99	1	0.99	0.50
Neurological symptom	97.81	0.97	1	0.98	0.50
Neuromuscular block prolonged	99.45	0.99	1	0.99	0.50
Neuromyopathy	99.45	0.99	1	0.99	0.50
Neuropathy	86.33	0.90	0.93	0.92	0.65
Neuropathy peripheral	80.32	0.86	0.90	0.88	0.58
Neurosis	95.0	0.97	0.97	0.97	0.63
Neurotoxicity	94.53	0.96	0.97	0.97	0.56
Optic neuritis	94.5	0.95	0.98	0.97	0.49
Peripheral motor neuropathy	98.90	0.98	1	0.99	0.50
Peripheral sensorimotor neuropathy	98.90	0.99	0.99	0.99	0.49
Peripheral sensory neuropathy	94.53	0.97	0.97	0.97	0.48
Polyneuropathy	96.72	0.97	0.98	0.98	0.49
Post herpetic neuralgia	100	1	1	1	1

Modelling using Merged features

	Accuracy (%)	Precision	Recall	F-score	AUC
Arteritic anterior ischaemic optic neuropathy	99.45	0.99	1	0.99	0.50
Autonomic neuropathy	99.45	0.99	1	0.99	0.50
Nervous system disorder	79.78	0.83	0.80	0.82	0.79
Neuralgia	85.79	0.93	0.90	0.92	0.61
Neuritis	90.71	0.93	0.96	0.95	0.58
Neuritis retrobulbar	99.45	0.99	1	0.99	0.50
Neuroleptic malignant syndrome	93.98	0.96	0.97	0.96	0.68
Neurologic reaction	99.45	0.99	1	0.99	0.75
Neurological impairment	99.45	0.99	1	0.99	0.50
Neurological symptom	96.72	0.97	0.98	0.98	0.49
Neuromuscular block prolonged	99.45	0.99	1	0.99	0.50
Neuromyopathy	99.45	0.99	1	0.99	0.50
Neuropathy	81.42	0.90	0.88	0.89	0.62
Neuropathy peripheral	80.87	0.88	0.88	0.88	0.64
Neurosis	93.98	0.97	0.96	0.96	0.69
Neurotoxicity	93.98	0.97	0.96	0.96	0.69
Optic neuritis	93.44	0.96	0.96	0.96	0.60
Peripheral motor neuropathy	98.36	0.98	0.99	0.99	0.49
Peripheral sensorimotor neuropathy	99.45	0.99	1	0.99	0.50
Peripheral sensory neuropathy	96.17	0.97	0.98	0.98	0.49
Polyneuropathy	97.81	0.97	1	0.98	0.50
Post herpetic neuralgia	99.45	1	0.99	0.99	0.99
